# Tau Acts in Concert With Kinase/Phosphatase Underlying Synaptic Dysfunction

**DOI:** 10.3389/fnagi.2022.908881

**Published:** 2022-05-27

**Authors:** Xing Fan, Liye Xia, Zheng Zhou, Yanyan Qiu, Chenhao Zhao, Xiaomin Yin, Wei Qian

**Affiliations:** ^1^Department of Biochemistry and Molecular Biology, Medical School, Jiangsu Key Laboratory of Neuroregeneration of Jiangsu and Ministry of Education of China, Co-innovation Center of Neuroregeneration, Nantong University, Nantong, China; ^2^NMPA Key Laboratory for Research and Evaluation of Tissue Engineering Technology Products, Nantong University, Nantong, China

**Keywords:** tau, kinase, phosphatase, synaptic dysfunction, Alzheimer's disease

## Abstract

Alzheimer's disease (AD) is characterized by two pathological features: neurofibrillary tangles (NFTs), formed by microtubule-associated protein tau, and abnormal accumulation of amyloid-β (Aβ). Multiple evidence placed synaptic tau as the vital fact of AD pathology, especially at the very early stage of AD. In the present review, we discuss tau phosphorylation, which is critical for the dendritic localization of tau and synaptic plasticity. We review the related kinases and phosphatases implicated in the synaptic function of tau. We also review the synergistic effects of these kinases and phosphatases on tau-associated synaptic deficits. We aim to open a new perspective on the treatment of AD.

## Introduction

Tau, a microtubule-associated protein, is important for microtubule assembly and stabilization (Weingarten et al., 1975; Drechsel et al., 1992). There are six isoforms of tau generated from alternative splicing of *MAPT* gene transcripts in the human brain (Goedert et al., 1989). It is widely believed that the majority of tau localizes in the axon, while the minority of tau is found in the soma and dendrites (Black et al., 1996; Mandell and Banker, 1996). For a long time, the role of tau is restricted to the establishment of neuronal polarity, axonal elongation, and transportation by its microtubule-associated ability (Caceres and Kosik, 1990; Esmaeli-Azad et al., 1994; Dixit et al., 2008). Evidence supports that tau is also located in dendrites and synapses of healthy neurons. Endogenous tau localized in dendrites and post-synapses was found both in rodent neurons (Ittner et al., 2010; Mondragon-Rodriguez et al., 2012; Zempel et al., 2013; Kimura et al., 2014; Swanson et al., 2017) and in mice brain (Xia et al., 2016). Physiological presynaptic and postsynaptic tau is also observed in human brains (Tai et al., 2012). Therefore, the post-synaptic localization of tau suggests a novel role of tau, contributing to postsynaptic signaling scaffolds and synaptic plasticity (Regan et al., 2017). Tau interacts with the postsynaptic density protein 95 (PSD-95)/N-Methyl-D-Apartate receptors (NMDARs) complex in the postsynapse, where tau stabilizes NMDARs to regulate synaptic plasticity (Ittner et al., 2010). External [for example, amyloid-beta (Aβ)] and intrinsic factors (for example, post-translational modification of tau) trigger increased concentration of tau in dendrites and post-synapses (mislocalization of tau) (Yin et al., 2021). Zempel et al. reported the missorting of hyperphosphorylated tau from the axon toward dendrites and spines (Zempel et al., 2010). Hyperphosphorylated tau mislocalized in the somatodendrites in AD leads to synaptic dysfunction (Ballatore et al., 2007; Hoover et al., 2010; Li et al., 2011). It also has been shown that tau plays a role in synaptic impairments (Polydoro et al., 2009). Teravskis et al. recently indicated that tau mislocalization to dendritic spines depends on site-specific phosphorylation of tau in the C-terminal domain and that P301L-induced tau mislocalization to dendritic spines can be prevented by the blockage of both GSK3β and CDK5 (Teravskis et al., 2021). Synaptic dysfunction is the earlier event than the extracellular amyloid plaques deposition of amyloid-β (Aβ) peptides and intracellular neurofibrillary tangles (NFTs), the two hallmarks of Alzheimer's disease (AD), and best correlates with cognitive deficits in brain of patients with AD (Yin et al., 2021). In this review, we focus on the phosphorylation profile of tau orchestrated by kinases and phosphatases in synaptic dendrite compartments. Controlling tau phosphorylation to prevent synaptic deficits and cognitive impairments through related kinases and phosphatases may be a viable therapeutic strategy.

## The Dendrite and Post-Synaptic Localization of Phospho-Tau

A series of evidence support that phosphorylation of tau directly contributes to dendrite and post-synaptic distribution of tau. Xia, D. et al. reported that single phospho-mimicking tau mutants at T231/S235, S262/S356, or S396/S404 trigger redistribution of tau into dendritic spines in neuronal cells (Xia et al., 2015). Phospho-mimicking tau with 14 simultaneous mutant sites promotes tau to dendritic spines in cultured neurons and subsequently leads to synaptic impairments (Hoover et al., 2010; Miller et al., 2014). Zempel et al. showed that targeting of tau into dendritic spines is dependent on phosphorylation of Tau at the KXGS-motifs (Zempel et al., 2013). In AD brain and transgenic mice, increased tau phosphorylation results in post-synaptic mislocalization of tau (Tai et al., 2012; Dejanovic et al., 2018). The direct pathological evidence revealed that mislocalization of tau to dendrites is an early event in AD pathogenesis (Hoover et al., 2010; Braak and Del Tredici, 2011; DeVos et al., 2018). Overall, the presence of phospho-tau in dendritic spines appears to be the early indicator of AD.

## Post-Synaptic Tau and Kinases

### Tau and GSK3

The GSK3 exists as two isoforms: GSK3α and GSK3β, both of which are prolific in the nervous system (Woodgett, 1990).

The GSK3β is a major pathogenic factor of AD (Takashima, 2006) and has abnormal activity in human AD brains (Leroy et al., 2007; DaRocha-Souto et al., 2012). Tau is regulated by many brain protein kinases (Martin et al., 2013), among which glycogen synthase kinase 3β (GSK3β) is the major Ser/Thr tau kinase.

The GSK3β is found in the postsynaptic compartment and is involved in the regulation of synaptic functions, including long-term synaptic plasticity (Peineau et al., 2007, 2009). Synaptic tau plays an important role in the regulation of long-term depression (LTD), a form of synaptic weakening (Kimura et al., 2014). GSK3β targets tau in the synaptic compartment (Llorens-Martin et al., 2013). However, GSK3β phosphorylates tau at Ser396, which is critical for LTD (Regan et al., 2015).

**Figure 1:**
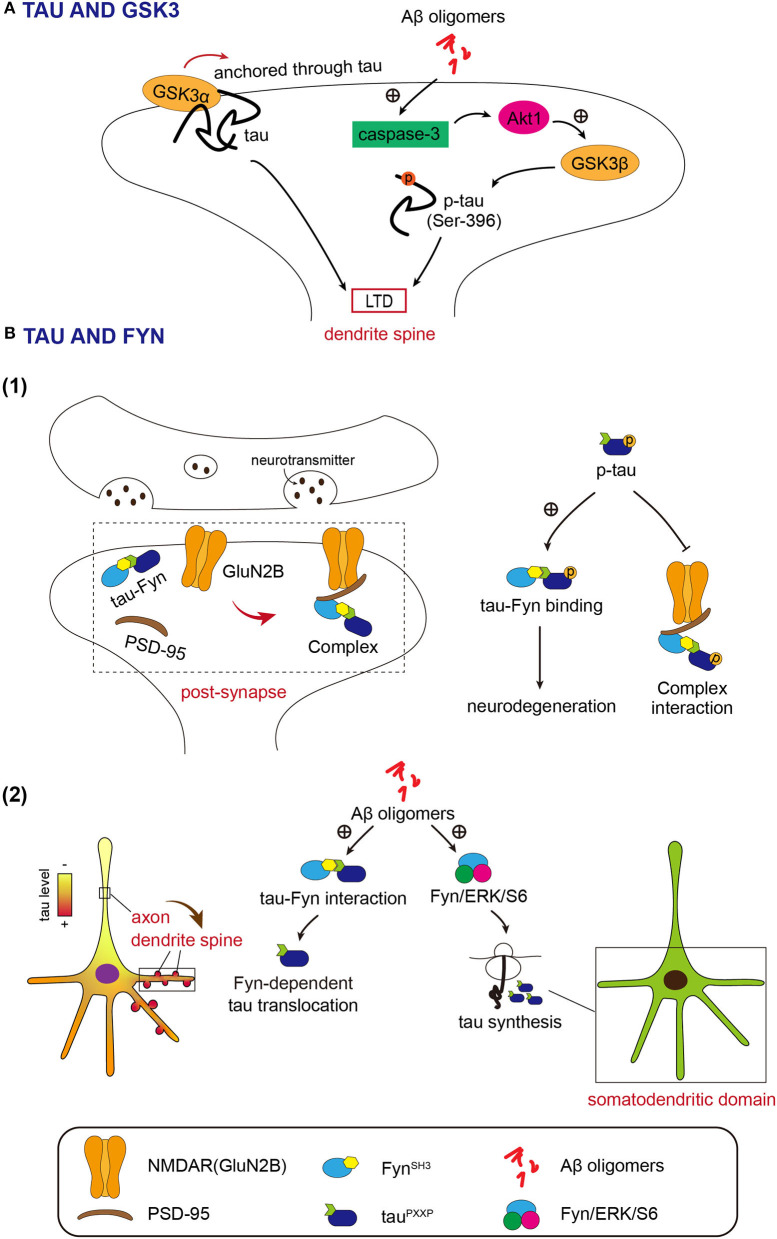
**(A)** Both GSK3α and GSK3β function in long-term depression (LTD) depends on tau. Activated GSK3β resulted from Akt1 cleavage by Aβ, phosphorylates tau in the synaptic compartment at Ser 396 to initiate LTD. GSK3α induces LTD by entrance into the dendrite spine temporarily *via* tau. **(B)** Fyn acts in the dendrite spine closely associated with tau. (1) Fyn-tau-PSD-95-NMDAR complex. Fyn interacts with tau *via* the SH3 domain and targets both PSD-95 and GluNR2B subunit of N-Methyl-D-Apartate receptors (NMDARs) by tau. Phosphorylation of tau promotes the tau-Fyn binding and meanwhile weakens the interaction between tau/Fyn and PSD-95/GluNR2B. (2) The intermediation of Aβ oligomer between tau and Fyn. Aβ oligomer enhances the interaction between tau and Fyn and leads to the dendrite spine translocation of tau in a Fyn-dependent manner. Aβ oligomer also simulates the Fyn/ERK/S6 pathway to enable de novo protein synthesis of tau in the somatodendritic domain.

Multiple evidence indicated that Aβ oligomers can induce the activation of neuronal caspase-3 (Marin et al., 2000; Garwood et al., 2011; Narayan et al., 2014), which leads to the Akt1 cleavage and GSK3β activation. Ultimately, the activated GSK3β will mediate phosphorylation of tau, which leads to pathological synaptic weakening in AD (Figure 1A; Lei et al., 2011).

It is considered that GSK3β contributes to most of the neuronal functions of GSK3, including those in synaptic plasticity (Maurin et al., 2013). This viewpoint was challenged by Draffin et al. They found that GSK3α, but not GSK3β, is required for LTD and is transiently recruited and anchored in dendritic spines through tau during LTD induction (Figure 1A; Draffin et al., 2021). Therefore, both GSK3β-mediated tau phosphorylation and dendritic spine anchor of GSK3α *via* tau may play a role in LTD.

### Tau and Fyn

The non-receptor-associated tyrosine kinase Fyn is one of eleven members of the Src family of protein-tyrosine kinases (Nygaard et al., 2014). Shirazi and Wood demonstrated elevated Fyn immunoreactivity in AD brain compared to controls (Shirazi and Wood, 1993). Evidence also supports that Fyn levels correlate with clinicopathological markers of neurodegeneration in AD (Ho et al., 2005).

The Src homology-3 (SH3) domains in Fyn interact with tau *via* Proline-X-X-Proline (PXXP) motifs in the Proline-rich region of tau (Lee et al., 1998; Ittner et al., 2010; Lau et al., 2016). In a tau-dependent manner, Fyn targets post-synapse, where Fyn binds to PSD-95, a key scaffolding protein for post-synaptic receptors, and NMDA receptors (Ittner et al., 2010; Mondragon-Rodriguez et al., 2012). The NMDA receptor subunit GluN2B, Fyn, tau, and the post-synaptic scaffolding protein PSD-95 form a complex (Figure 1B; Ittner et al., 2010; Mondragon-Rodriguez et al., 2012). GluN2B is phosphorylated by Fyn at Y1472, which regulates the content of synaptic NMDA receptors and increases NMDA receptor-dependent synaptic currents (Roche et al., 2001; Lavezzari et al., 2003; Salter and Kalia, 2004). Phosphorylation of GluN2B by Fyn enhances the interaction between NMDAR and PSD-95 in synapses (Ittner et al., 2010). In AD animal models, stabilization of the NMDAR/PSD95 complex is associated with Aβ-induced excitotoxicity (Ittner et al., 2010). Reports showed that tau-Fyn interaction is increased upon phosphorylation of tau protein (Bhaskar et al., 2005; Mondragon-Rodriguez et al., 2012). On the other hand, the association of tau/Fyn with PSD-95 and GluN2B is decreased depending on the phosphorylation of tau (Mondragon-Rodriguez et al., 2012). Research results also suggest that phosphorylation of tau at threonine 231 may be important for this post-synaptic protein interaction (Mondragon-Rodriguez et al., 2012). Investigations into the impact of FTDP-17 (frontotemporal dementia and Parkinsonism linked to chromosome 17) mutations on the Fyn-tau interaction provide evidence that the abnormal interaction of tau-Fyn results in the neurodegenerative process (Figure 1B; Lee et al., 2004; Bhaskar et al., 2005; Ittner et al., 2011).

The Aβ oligomers promote tau-SH3 interactions and cause tau translocation into dendritic spines (Frandemiche et al., 2014). Findings suggest that increased dendritic tau levels in AD come from growing dendritic tau translation induced by Aβ in a Fyn-dependent manner (Li and Gotz, 2017). Oligomeric Aβ activates Fyn/ERK (extracellular regulated protein kinases)/S6 signaling pathway in the somatodendritic compartment and mediates *de novo* protein synthesis of tau in the somatodendritic domain (Figure 1B; Li and Gotz, 2017). This report revealed the new pathogenesis of AD.

Generally, tau and Fyn may have a mutual effect on each other in the NMDAR complex to regulate synaptic function downstream of Aβ oligomers.

Existing research results also provide some clues about other kinases interplaying with tau in synaptic function as shown in Table 1.

**Table 1:** Post-synaptic tau and other kinases.

**Kinases**	**Interplay with post-synaptic tau**	**References**
ERK	Tau depletion leads to activation of ERK following extrasynaptic NMDA receptor stimulation	Sun et al., 2016
	Post-synaptic tau in complex of PSD-95 regulates NMDAR-mediated ERK activation via SynGAP1	Bi et al., 2017
JNK	JNK is the mediator of tau induced synapse loss and tau related mature synapses maintenance	Voelzmann et al., 2016
p38γ MAPK	p38γ MAPK phosphorylates tau at Threonine-205 (T205), a site-specific phosphorylation that mediates a protective function of tau	Ittner et al., 2016
	Phosphorylation of tau by postsynaptic p38γ alleviates Aβ-induced excitotoxicity and hence ameliorates memory deficits	Ittner et al., 2020
PKA	Intracellular accumulated tau inhibits PKA, resulting in synaptic and memory impairments	Ye et al., 2020
CDK5	Abnormal activity of CDK5 leads to hyperphosphorylation of tau, the loss of dendritic spines and impairments of synaptic plasticity in AD	Kimura et al., 2014
JAK2	hTau accumulation induces JAK2/STAT1-mediated suppression of NMDAR expression, which results in impairments of synaptic plasticity and memory deficits	Li et al., 2019

*ERK, extracellular regulated protein kinases; JNK, Jun N-terminal kinase; p38γ MAPK, p38γ mitogenactivated protein kinase; PKA, protein kinase A; CDK5, cyclin dependent kinase 5; JAK2, Janus kinase 2*.

## Post-Synaptic Tau and Phosphatase

### Tau and PP2A

**Figure 2:**
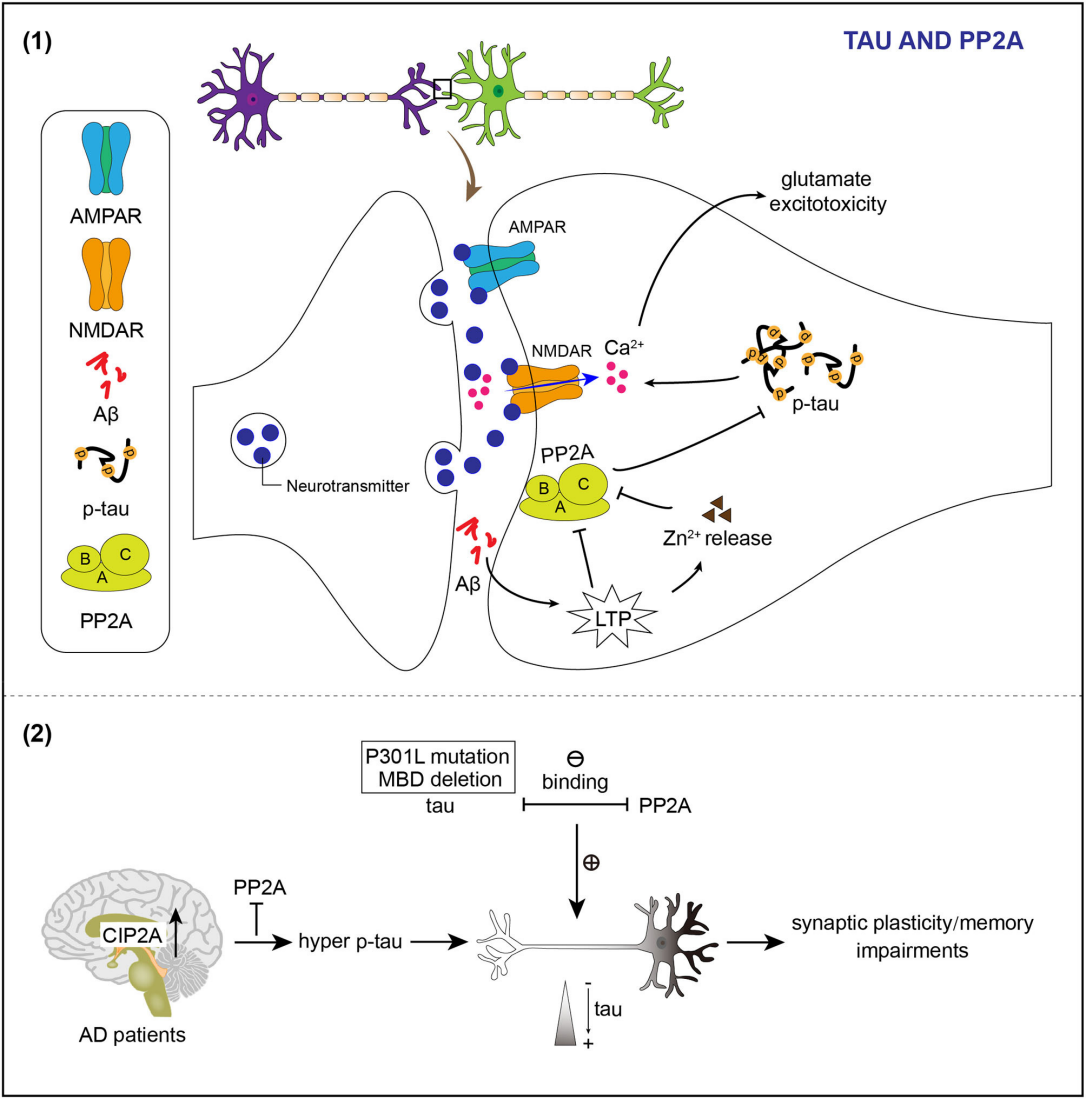
PP2A regulates phosphorylation and the location of tau in neuronal dendrites. (1) Induction of LTP by Aβ causes the release of Zn^2+^ to inhibit the activity of PP2A and therefore results in hyperphosphorylation of tau. Abnormal post-synaptic regulation of tau and PP2A contributes to the accumulation of phosphorylated tau, which enhances NMDAR-dependent Ca^2+^ influx and promotes glutamate excitotoxicity. (2) Mutation of tau impairs the tau/PP2A binding and thereby leads to dendrite spine location of tau. Reduced activity of PP2A attributing to the increased endogenous inhibitor of PP2A in the brains of patients with Alzheimer's disease (AD) facilitates hyperphosphorylation of tau and then the mislocation of tau in the dendrite spine, which relates to impairments of synaptic plasticity and memory deficits.

The Ser/Thr protein phosphatase 2A (PP2A) is an important phosphatase of tau in the brain (Sontag et al., 1996; Liu et al., 2005). PP2A is downregulated in the AD brain, which appears to contribute to tau hyperphosphorylation found in AD (Gong et al., 1993, 1995; Liu et al., 2005). PP2A consists of a catalytic C, a scaffolding A, and a regulatory B subunit as a heterotrimeric complex (Virshup and Shenolikar, 2009). PP2A holoenzymes can directly interact with tau (Sontag et al., 1999, 2012; Xu et al., 2008). The interaction between PP2A and tau is modulated by a specific RTPPKSP Proline-rich motif in tau (Sontag et al., 2012). PP2A localizes to neuronal dendrites and its activity decreases upon LTP induction (Fukunaga et al., 2000). Abnormal induction of LTP by Aβ may inhibit PP2A activity, resulting in hyperphosphorylation of dendritic tau (Sontag and Sontag, 2014). Synaptically released Zinc modulates synaptic transmission and plasticity *via* interacting with ion channels, receptors, and transporters (Karakas et al., 2009; Takeda et al., 2010). Zinc released by increased synaptic activity induces tau hyperphosphorylation through PP2A inhibition (Figure 2; Sun et al., 2012).

The PP2A also exists in PSDs and synaptic plasma membrane fractions to regulate the phosphorylated state of dendritic tau (Chan and Sucher, 2001). The pathological accumulation of phosphorylated tau caused by aberrant post-synaptic regulation of tau and PP2A enhances NMDAR-dependent Ca^2+^ influx and promotes glutamate excitotoxicity (Figure 2; Haass and Mandelkow, 2010).

The PP2A is also implicated in controlling tau subcellular distribution. The microtubule-binding domain deletion or P301L mutations in tau weakens the binding of PP2A to tau and makes tau localize to dendritic spines (Xia et al., 2015).

The level of cancerous inhibitor of PP2A (CIP2A), an endogenous PP2A inhibitor, is upregulated in brains of patients with AD (Shentu et al., 2018). Overexpression of CIP2A inhibits PP2A, which leads to tau hyperphosphorylation and tau mislocalization to dendrites and spines (Figure 2; Shentu et al., 2018).

### Tau and Calcineurin

Tau and calcineurin are described as two signaling molecules in Aβ-initiated synaptic dysfunction early in AD. AMPAR signaling deficits, induced by Aβ_1−42_ oligomer, require both phosphorylations of tau and activation of calcineurin. Mislocalization of tau to dendritic spines causes activation of calcineurin (Miller et al., 2014).

Tau accumulation disrupts intracellular calcium signaling, leading to cAMP-response element binding protein (CREB) inactivation by calcineurin activation to suppress the expression of synaptic proteins (Yin et al., 2016).

The entirety of tau phosphorylation and its related tau kinases and phosphatases in synaptic dysfunction is still largely unknown, and the research needs to be on-going.

## Synergic Network of Kinases/Phosphatases in Synapse

Multiple evidence supports that synergistic interactions exist among kinases and phosphatases to regulate synaptic functions. GSK3β acts as a molecular switch between NMDAR-LTP and NMDAR-LTD (Peineau et al., 2007). GSK3 has been discovered to be a key regulator of synaptic plasticity (Bradley et al., 2012). GSK3 is a main component of a complex phosphorylation cascade involved in synaptic plasticity. Several kinases such as extracellular regulated protein kinases (ERK), the dual-specificity tyrosine kinase ZAK1, mitogen-activated protein kinase 1/2 (MEK1/2), proline-rich tyrosine kinase 2 (Pyk-2), and the non-receptor-associated tyrosine kinase Fyn has been reported to interact with GSK3 and regulate its function (Kim et al., 1999, 2002; Lesort et al., 1999; Hartigan et al., 2001; Ding et al., 2005). The phosphorylation of GSK3β at Tyr216, necessary for its activity (Hughes et al., 1992, 1993), may be regulated by Fyn (Lesort et al., 1999) or Pyk2 (Hartigan et al., 2001). Phosphorylation of GSK3β at Ser9 inhibits its basal enzymatic activity (Doble and Woodgett, 2003). Several signaling pathways are involved in controlling the Ser9 phosphorylation state of GSK3β. For example, insulin causes the inactivation of GSK-3 through the phosphatidylinositide (PI) 3-kinase (PI3K)- protein kinase B (PKB; also termed Akt) pathway. Insulin binds to its receptor to trigger the phosphorylation and the plasma membrane recruitment of Insulin receptor substrate (IRS) proteins. Then the tyrosine-phosphorylated IRS proteins recruit PI3K to the membrane, where it produces PtdIns (3,4,5) P3 (PIP3), the second messenger. PIP3 binds to pyruvate dehydrogenase kinase 1 (PDK1) and Akt, and co-localizes them at the plasma membrane. Subsequently, PDK1 activates Akt. Active Akt then inhibits GSK3 by phosphorylating Ser21 (GSK3α) and Ser9 (GSK3β) (Cross et al., 1995; Cohen et al., 1997). The Akt pathway regulates the phosphorylation of GSK3β during LTP (Peineau et al., 2007). A recent study showed that sulfhydration of Akt, detected in the postmortem brains of patients with AD, inhibits its interaction with GSK3β and subsequently decreases the Ser9 phosphorylation of GSK3β, then, the activated GSK3β promotes tau phosphorylation and cognitive dysfunction (Sen et al., 2020). CaMKII (Song et al., 2010), protein kinase C (PKC) (Espada et al., 2009), PKA (Li et al., 2000; O'Driscoll et al., 2007; Shelly et al., 2010, 2011), PrkG1 (Zhao et al., 2009), p90 ribosomal protein S6 kinase (RSK) (Valerio et al., 2006), and Integrin-linked kinase (ILK) (Naska et al., 2006) also phosphorylate and inhibit GSK3. ILK phosphorylates Akt at serine-473, required for Akt activation, to inhibit GSK3β indirectly in addition to its direct phosphorylation of GSK3β (Delcommenne et al., 1998). Phosphorylation of GSK3β at Ser389 (Thr390 in humans) by p38 MAPK has been found to inhibit its activity (Thornton et al., 2008). The protein phosphatase 1 (PP1) dephosphorylates GSK3β at Ser9, which is the pathway to stimulate GSK3β activity in neurons (Bennecib et al., 2000; Morfini et al., 2004). After NMDA receptors stimulation, research reports have identified a positive feedback loop between PP1 and GSK3β, which means that GSK3β increases phosphorylation of inhibitor-2 (I-2) to activate PP1, whereas PP1 dephosphorylates GSK3β at Ser9 to further activate GSK3β (Zhang et al., 2003; Szatmari et al., 2005). During NMDAR-LTD, Ca^2+^ enters through NMDARs and binds to calmodulin. Then, Ca^2+^/calmodulin-dependent protein phosphatase (calcineurin, also termed as PP2B) is activated and dephosphorylates Inhibitor 1 (I-1), which finally causes activation of PP1 and contributes to the generation of LTD (Mulkey et al., 1994).

The tau-dependent recruitment of Fyn to post-synaptic NMDAR complexes is crucial for Aβ-induced excitotoxicity (Ittner et al., 2010). Post-synaptic tau control NMDAR-mediated ERK activation by limiting the binding of SynGAP1 to PSD-95 in post-synaptic NMDAR complexes (Bi et al., 2017). Aβ induces phosphorylation of tau by activating GSK3β in an NMDAR-dependent manner (Tackenberg et al., 2013). Taken together, Fyn, ERK, and GSK3β are downstream factors of post-synaptic tau in complex with PSD-95. Fyn inhibits the interaction between PP2A and tau (Sontag et al., 2012). On the contrary, reduced methylation and levels of PP2A in AD will disrupt normal PP2A-tau interactions and enhance the binding of Fyn kinase to the tau proteins (Sontag et al., 2007). Fyn-SH3 interacts more tightly with 3R- than 4R-Tau (Bhaskar et al., 2005), while PP2A prefers 4R- than 3R-Tau (Sontag et al., 1999). Since Fyn functions downstream of insulin and binds to insulin receptor substrate 2 (IRS-2) to regulate LTP, Aβ oligomers induce abnormal glutamatergic synaptic transmission and LTP/LTD through interaction between somatodendritic tau and Fyn, which is associated with PI3K/Akt signaling (Martin et al., 2012).

## Therapeutic Target to Kinase/Phosphatase Acting on Post-Synaptic Tau

Since Aβ has been demonstrated to be the primary factor in the pathogenesis of AD over the years, the therapy strategies for AD have been focused on alleviating Aβ pathology. However, tau pathology correlates much better with the cognitive decline in AD than Aβ plaques (Bejanin et al., 2017; Lowe et al., 2018). Therefore, the therapeutic approach for AD has been progressively turned to tau pathology, especially synaptic dysfunction attributed to synaptic mislocalized tau, which is the early event in the brain of patients with AD.

Given that GSK3β exists in the post-synaptic compartment and the interplay between GSK3β and tau is crucial for synaptic function, GSK3 becomes a charming therapeutic target in AD. Although multiple GSK3 inhibitors have been developed over the years (Eldar-Finkelman and Martinez, 2011), almost none of them have been applied clinically. It remains a challenge to discover novel drugs, used as GSK3 inhibitors, to effectively treat AD. Anti-phospho-tau antibodies to GSK3 sites on tau are once applied to immunotherapy for AD, such as PHF-1 antibody (Boutajangout et al., 2011; Liu et al., 2016), targeting epitope pSer396/pSer404 of tau (Boutajangout et al., 2011; Liu et al., 2016), antibodies JNJ-63733657, PT3 (Chai et al., 2011; d'Abramo et al., 2013) and its humanized version, hTP3 (Van Kolen et al., 2020), targeting epitope pThr212/pThr217, and antibody PHF-13 (Sankaranarayanan et al., 2015) targeting epitope pThr231 and pSer396. Mouse models, used to test the therapeutic effect of these antibodies, show that tau pathology and functional deficits are reduced by immunotherapies (Bittar et al., 2020). Tau plays multiple roles including myelination, glucose metabolism, axonal transport, microtubule dynamics, iron homeostasis, neurogenesis, motor function, learning and memory, neuronal excitability, and DNA protection (Kent et al., 2020). Further efforts are still needed to obtain safe and efficient therapeutic approaches, which target only pathological tau but not physiological tau.

The SH3-containing protein Fyn interacts with tau, which is promoted by the Aβ oligomer. Aβ oligomer also causes dendritic translation of tau mediated by Fyn (Li and Gotz, 2017) and dendritic spine translocation of tau (Frandemiche et al., 2014). Travis Rush et al. targeted tau-Fyn interaction to investigate novel therapeutic strategies for AD. A peptide inhibitor is developed to alleviate Aβ oligomer toxicity through inhibiting tau-SH3 interactions, suggesting the therapeutic potential of inhibiting tau-SH3 interactions to treat AD (Rush et al., 2020). Miren Ettecho and colleagues found that inhibition of Fyn by masitinib could efficiently ameliorate synaptic dysfunction and dendritic spine aberrances induced by Aβ and tau in AD (Ettcheto et al., 2021).

As mentioned above, p38 MAPK can phosphorylate tau at specific sites associated with tau-mediated memory deficits. Studies showed that inhibition of p38 MAPK to normalize tau function is a potential therapeutic approach, eliminating the synaptic dysfunction in AD. A selective p38α MAPK direct inhibitor 8 has been reported to suppress the activity of p38α MAPK and, therefore, decrease tau phosphorylation and restrain cognitive impairment in aged hTau mice (Watterson et al., 2013).

The PP2A regulates phosphorylation of tau in neuronal dendrites and participates in synaptic function. Developing therapeutic approaches, which target PP2A and resist tau pathology in AD, are very important. Some clinically used drugs can activate PP2A to reduce tau phosphorylation, such as sodium selenate (van Eersel et al., 2010), memantine (Chohan et al., 2006), metformin (Kickstein et al., 2010), and so on. Since the broad effect of PP2A, there are multiple factors to be considered. It is not clear what primary mechanism of PP2A dysfunction should be preferentially targeted in clinical trials, since PP2A regulation is multisided and complex. Furthermore, specificity and side-effects are a huge issue due to the broad spectrum of PP2A. From animal tests to clinical trials in humans, there is still a long way to go.

## Conclusion

The present review discusses the phosphorylation modification of tau, the major factor related to the somatodendritic compartment and post-synaptic localization of tau. Both kinases (such as GSK3 and Fyn) and phosphatases (such as PP2A and calcineurin) regulate the synaptic localization of tau. Kinases/phosphatases act as a linkage between Aβ oligomer and tau to progress Aβ-initiated synaptic toxicity *via* synaptic translocation of tau. Dysregulation of kinases/phosphatases on tau triggers missorting of tau to dendrites and post-synapses and results in synaptic dysfunction. Synaptic tau implicates in LTD regulation, which is also controlled by kinases/phosphatases associated with synaptic tau. Kinases and phosphatases work together to regulate the localization of tau and synaptic plasticity. Thus, kinases and phosphatases, acting on synaptic tau, might be prospective therapeutic targets in AD.

## Author Contributions

WQ wrote the draft of the manuscript. All authors contributed to the manuscript revisions and as well as read and approved the submitted version.

## Funding

This work was supported in part by Nantong University, the Priority Academic Program Development of Jiangsu Higher Education Institution (PAPD), and grants from the National Natural Science Foundation of China (81872875, 81170317, and 81473218 to WQ and 81503077 to XY).

## Conflict of Interest

The authors declare that the research was conducted in the absence of any commercial or financial relationships that could be construed as a potential conflict of interest.

## Publisher's Note

All claims expressed in this article are solely those of the authors and do not necessarily represent those of their affiliated organizations, or those of the publisher, the editors and the reviewers. Any product that may be evaluated in this article, or claim that may be made by its manufacturer, is not guaranteed or endorsed by the publisher.
